# Gamified Physical-Digital Smoking Cessation Intervention for Young Adults: Mixed Methods Development and Usability Study

**DOI:** 10.2196/72749

**Published:** 2025-09-19

**Authors:** Natalia Bartłomiejczyk, Alessio De Santo, Adrian Holzer

**Affiliations:** 1 University of Neuchâtel Neuchâtel Switzerland; 2 HEG Arc HES-SO // University of Applied Sciences and Arts Western Switzerland Neuchâtel Switzerland

**Keywords:** smoking cessation, ambient intelligence, gamification, user study, behavioral research

## Abstract

**Background:**

Smoking remains a leading cause of death worldwide, with young adults particularly at risk due to the lack of targeted cessation initiatives. While mobile apps show promise in supporting smoking cessation, they primarily target smokers already motivated enough to install them, highlighting the need for interventions that reach those who are not yet ready to take that step.

**Objective:**

This paper focuses on designing and evaluating Smokwit, a digital smoking cessation intervention aimed at young adults during the act of smoking. Smokwit seeks to investigate the early stages of smoking cessation (precontemplation and contemplation) that are important yet rarely investigated.

**Methods:**

The paper is based on the design science research methodology where a digital intervention—Smokwit—was designed and evaluated in the wild using a mixed method approach combining quantitative results of a quasi-experiment with qualitative insights from users and experts. More specifically, Smokwit is a novel gamified ambient intervention that integrates a connected ashtray with a mobile app. The ashtray aims to trigger processes of change, in particular consciousness raising and social liberation (as part of the transtheoretical model of change) by provoking curiosity, self-reflection, and ad-hoc peer discussions among smokers. The linked mobile app is designed to reinforce this goal by providing smoking cessation self-help material and coaching possibilities. We evaluated the effectiveness of this intervention through a 3-month field study designed as a quasi-experiment with a treatment and control group (n=46). A qualitative analysis with users (n=10) and smoking cessation experts (n=7) provides insights into the type of interactions that happened within and outside the system as well as practical implications for smoking cessation organizations.

**Results:**

The qualitative findings revealed that the intervention promoted smokers’ self-reflection, peer discussions, and mobile app interactions. Furthermore, the quantitative analysis uncovered a possible trend toward increased readiness to quit among smokers in the treatment group compared to the control group; however, this did not reach conventional levels of statistical significance (b=1.33; *z*=1.91; *P*=.06).

**Conclusions:**

Smokwit provides encouraging insights into how to design a bottom-up digital intervention that targets young adults at an opportune moment to support them on their smoking cessation journey.

## Introduction

### Background

Despite many years of prevention, tobacco addiction is still the cause of approximately 8 million deaths annually, making it the foremost preventable cause of death globally [1]. Efforts to pass comprehensive laws and regulations can be hindered in countries where the tobacco industry provides economic benefits and thus wields significant lobbying power. For example, in Switzerland, where the industry has some of its major headquarters, this lobbying power is particularly strong [2]. Specifically, the tobacco industry in Switzerland contributes approximately US $7 billion in annual revenue, representing about 1% of the country’s gross domestic product, and provides employment for around 11,500 people (0.2% of the labor force) [3]. However, it can be argued that these economic benefits are offset by the health-related expenses of US $11 billion yearly [4], including the 10,000 smoking-related deaths annually [5]; thus, passing top-down regulation remains highly challenging. As a result, in such countries, it is crucial to devise effective bottom-up prevention measures to support smoking cessation.

There are effective evidence-based interventions that could be helpful for young smokers; these include brief advice, behavioral support, pharmacotherapy, and abstinence evaluation [6]. Furthermore, digital technologies, such as mobile apps, have been identified as a potentially effective, lightweight, and scalable complementary support for smoking cessation [7-9]. These technologies could represent a particular interest, potentially lowering current funding, time, and human resource barriers for health promotion [10]. For instance, there is a wide range of specialized mobile apps that could act as catalysts to provide this support [9,11]. However, despite their potential benefits, there remains a challenge with user engagement, especially when the apps are not well-designed or integrated into clinical or daily workflows [12]. Indeed, even minor frictions prevent smokers from engaging in effective behavior change interventions [13]. For instance, it is challenging to get a smoker to seek digital self-help information, sign up for peer support, or even register for face-to-face counseling [14]. As such, one of the main challenges in smoking cessation interventions is effectively getting smokers on board [15,16]. Most existing digital solutions require individuals to first be aware of the app’s existence, and then take the additional steps of downloading and installing it; there are barriers that can deter engagement, especially for those not actively seeking help. Conversely, our approach initiates onboarding directly at smoking hotspots through physical devices, offering immediate and contextual engagement. This paper aims to address this gap by exploring an alternative, low-threshold entry point into cessation support.

A particularly important demographic to target prevention measures are young adults (ages 18 to 35 years). Most smokers start early, with 87% beginning before the age of 21 years [17], and young adults often represent the largest smoking demographic [17]. This is particularly problematic because the earlier one starts smoking regularly, the faster addiction sets in [18], thus making it harder to quit. Despite approximately half of young people wishing to quit [19], only an estimated 5% manage to do so during their youth [20]. Furthermore, smoking cessation initiatives that are specifically aimed at young adults are lacking [21]. As such, we seek to address the following research question: How can a digital intervention be designed to increase young adult engagement with evidence-based smoking cessation interventions?

### Contributions

This research makes 3 main contributions to the literature on digital interventions for smoking cessation. First, it introduces Smokwit—a novel digital system specifically designed to reach smokers at physical smoking hotspots—immediately before, during, or after the act of smoking. Unlike conventional tools that rely on user motivation or active digital engagement, Smokwit reduces entry barriers by engaging users in situ and at opportune moments [22,23]. It leverages connected ashtrays as ambient objects [24], facilitating a seamless transition from the physical environment to digital smoking cessation resources. Furthermore, Smokwit is conceptualized as a motivational affordance [25], a system intentionally designed to trigger behavioral outcomes by incorporating gamification mechanisms such as competition and rewards [26], with the goal of increasing engagement with evidence-based interventions. Second, this paper presents a real-world validation of the Smokwit system through a 3-month quasi-experimental field study (n=46) conducted across 2 university campuses. This study combines quantitative data (surveys and system logs) with qualitative insights from interviews with participants (n=10) and experts (n=7), thus providing a comprehensive understanding of system engagement and user experience in a natural context. Third, the paper contributes empirical insights into the early stages of smoking cessation, specifically the precontemplation and contemplation stages as defined by the transtheoretical model (TTM) of health behavior change [27]. These stages are critical yet underexplored in digital health research [28]; our findings shed light on how low-friction, context-aware interventions can effectively engage smokers who are not yet actively seeking help.

### Smoking Cessation Process

Giving up smoking is more of a process rather than a single decision or action [29]. A useful theoretical lens for analyzing this process is the TTM [27], which conceptualizes behavior change as a progression through discrete stages. Unlike continuum models, which assume interventions can be applied at any point, stage models such as the TTM emphasize that different strategies are appropriate at different stages in the change process [29-31]. In this context, the stages toward smoking cessation can be seen as a smoker’s readiness to quit (RTQ). TTM hypothesizes that this change occurs in 6 distinct steps, also called stages of change:

- Stage 1 – precontemplation: no intention of taking action within the next 6 months.
- Stage 2 – contemplation: intention of taking action within the next 6 months.
- Stage 3 – preparation: intention of taking action within the next 30 days; thus, some behavioral steps have already been taken.
- Stage 4 – action: changed overt behavior for less than 6 months.
- Stage 5 – maintenance: changed overt behavior for more than 6 months.
- Stage 6 – termination: no temptation to relapse and 100% confidence.

In this paper, we focus on the early stages—precontemplation and contemplation—that are especially underaddressed in digital health interventions. These are stages where smokers are not yet ready to take direct action; however, they may be more receptive to change through targeted support. According to TTM, progress between these stages is mediated by cognitive and affective processes such as raising consciousness, meaning increasing awareness through information and feedback, and social liberation, thus reinforcing social norms that support healthy behavior [31].

Our intervention, Smokwit, is deliberately designed to activate these processes in contextually appropriate ways. By placing connected ashtrays at smoking hotspots, Smokwit raises awareness of quitting resources (consciousness raising) and introduces quitting as a socially supported behavior (social liberation). The use of ambient technology lowers friction for engagement, helping reach users who are not yet actively seeking help. Moreover, Smokwit incorporates gamification elements, such as rewards and peer comparison, to further reinforce social motivation and self-reflection [25,26]. In line with TTM, these mechanisms aim to help users move from passive awareness (stage 1) to the contemplation of change (stage 2).

In addition to these motivational design elements, Smokwit supports 2 evidence-based engagement strategies. First, peer interaction is integrated into the system to support social comparison and group influence, which have been shown to strengthen motivation and behavior change in stopping smoking [6,32,33]. This leads to our first premise:

- Premise 1: Engagement with peer groups helps smokers increase their RTQ.

Second, Smokwit offers access to curated self-help material, which can also support users in the early stages of change. Prior studies on smoking cessation apps show that such content can improve RTQ, especially among less motivated users [9,34-37]. This confirms our second premise:

- Premise 2: Engagement with self-help material helps smokers increase their RTQ.

By aligning Smokwit’s design with key TTM processes and stages, we aim to offer a context-sensitive intervention that addresses a critical gap in digital smoking cessation efforts.

### Gamifying Smoking Cessation Interventions

There is a growing number of technological solutions, such as forums and mobile apps, that give smokers access to self-help material and leverage peer support by facilitating multilateral interactions between smokers on dedicated social media channels [6,9,38,39]. However, it is unclear how many smokers actually engage with such resources [9]. A particular challenge is to capture smokers who are still in the precontemplation phase and, as such, are not interested in quitting at present and to engage for the first time.

One approach to increase engagement with digital or mobile content is to conceptualize interventions as motivational affordances, such as gamification or digital nudging [40]. These are artifacts designed to foster specific behaviors [25]. An immediate behavioral outcome in our case would be increased engagement with digital self-help resources or peer group reflection. A distal outcome would be to move smokers from the precontemplation to the contemplation stage by increasing their RTQ.

The gamification approach [26], where game elements such as points, leaderboards, or badges are integrated into nongame systems, has already been extensively used in mobile apps targeting smoking cessation [9]. Typically, around 65% of the top 100 smoking cessation apps include some kind of reward system, such as points or digital badges and trophies awarded when milestones are reached (eg, number of days without smoking) [9]. However, these systems primarily use these mechanisms to encourage existing users to stay on the platform and are not designed to engage new users. In this paper, we want to address this gap by focusing on bringing new users to the system.

### Opportune Moment Prompting

One approach to bringing users to the system can be inspired by digital nudging—a motivational affordance approach that modifies a user’s choice to make certain behaviors more attractive [41]. The idea with certain types of digital nudges is to prompt users at opportune times to transition from one context to another [23,42]. An example of such prompting is simply to display push notifications on the phone, which indicate to users that something is happening, for example, a message has been received, a location reached, or a time limit exceeded. The opportune timing of prompting is an important aspect [22]. These moments can be life events, where stopping smoking could be linked to a health-related event such as a doctor’s visit, pregnancy, or hospitalization [43]. However, there could also be moments close to more mundane behavior. For instance, road safety, reminding drivers to use their seat belts shortly before they drive away, significantly increases seat belt compliance in comparison to reminding drivers 5 minutes before they drive away [44]. In the smoking context, a recent study found promising results for face-to-face interventions in areas where smokers gather to smoke (smoking hotspots) [45]. In this paper, we build on these insights to investigate how a digital artifact could prompt users at smoking hotspots without the need for in-person interventions.

### Ambient Transition Device

**Figure 1:**
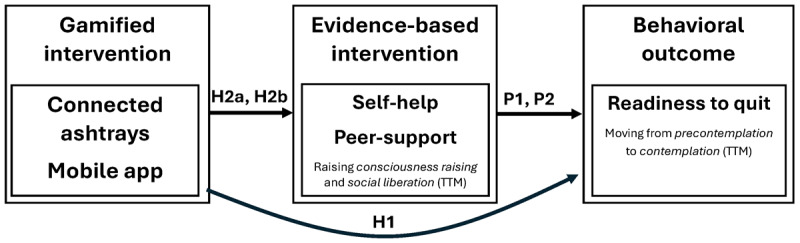
Overview of the full conceptual model illustrating our hypotheses (H1, H2a, and H2b) and premises (P1 and P2). TTM: transtheoretical model.

A promising approach to transition from a physical context, smoking, to a digital context, accessing digital content, is the use of ambient devices. An ambient device is a regular object from physical space augmented through a digital artifact, which can enhance awareness of digital activities [46]. In a knowledge-sharing system, a connected aquarium was designed to visualize digital contributions to motivate users to log in and make their own contributions [24]. Similarly, in the energy-saving context, artistic screens connected to smart meter data have been shown to increase awareness of energy consumption and encourage energy-saving behaviors [47]. In the medical domain, researchers provided patients with asthma with an “Asthma Buddy” teddy bear [48]. In this system, simple feedback is provided through the teddy bear, and more elaborate data are displayed on a mobile app. We build on these previous findings to hypothesize that a gamified ambient device at a hotspot could help reduce the friction for smokers to engage more with digital self-help and peer support. This increased engagement will lead to progress in the process of stopping smoking such as RTQ. Figure 1 illustrates our conceptual model. The gamified intervention, implemented through connected ashtrays in public smoking areas, acts as a playful and tangible nudge toward 2 core evidence-based strategies, self-help and peer support. We state our main hypothesis as follows:

- Hypothesis 1: Gamifying an ambient device at smoking hotspots increases RTQ.

We argue that this increase can happen by increasing engagement with self-help material and reflection among peers. Formally stated:

- Hypothesis 2a: Gamifying an ambient device at smoking hotspots increases engagement with self-help material.
- Hypothesis 2b: Gamifying an ambient device at smoking hotspots increases engagement with peers.

## Methods

### Overview

This research uses a user-centered design approach [49], which incorporates elements of design thinking [50] and design science research methodology [51,52]. The artifact’s design was guided by practice and theory, with psychological and behavioral outcomes used to measure its effectiveness. This project involved an 18-month collaborative effort with a multidisciplinary team of human-computer interaction and information systems researchers, embedded systems engineers, and partners from smoking cessation organizations. Co-design workshops were conducted with these partners to understand the issue and explore the design space before creating initial prototypes. Then the design was evaluated in the wild in a field study. The study takes a mixed method approach [53], combining elements from a quantitative quasi-experimental design setup with qualitative interview analyses. We adopted a multimodal data collection approach [54], with survey data, system logs, and interview transcripts.

### Ethical Considerations

The regional ethics committee of the Canton of Vaud, Switzerland (CER-VD) reviewed the study and determined that it fell outside the scope of the Swiss Human Research Act (Req-2022-01567); thus, no formal ethics approval was required. At the beginning of the survey, participants were informed about the purpose of the study, the voluntary nature of participation, and their right to withdraw at any time without consequences. Informed consent was implied by clicking “Next” to begin the survey. Data were pseudonymized, stored securely on access-restricted servers, and reported only in aggregate. No direct financial compensation was provided; participants completing both surveys were entered into a prize draw, while interviewees received no compensation.

### Smokwit System Design

#### Overview

To help young smokers in the precontemplation phase to increase their RTQ, Smokwit leverages two evidence-based behavioral support approaches directly at the smoking hotspot: (1) self-help material and (2) peer group support. The first approach, self-help material such as digital videos, can aid smokers without needing outside assistance, and it is deemed useful if it is tailored to individual smokers [55]. In the second approach, which may be more effective [56,57], peers provide mutual support and encouragement, either through a digital forum [11] or during in-person group sessions.

**Figure 2:**
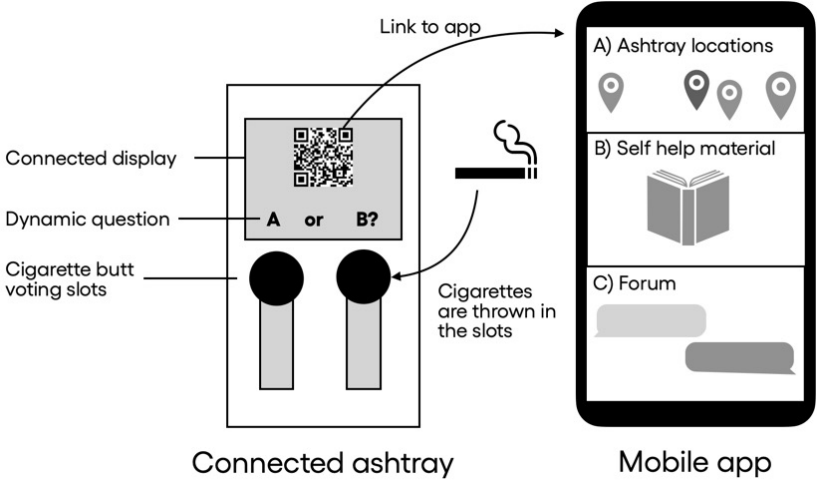
The Smokwit system design concept includes the connected voting ashtray with a link redirecting users to a mobile app with digital support materials.

**Figure 3:**
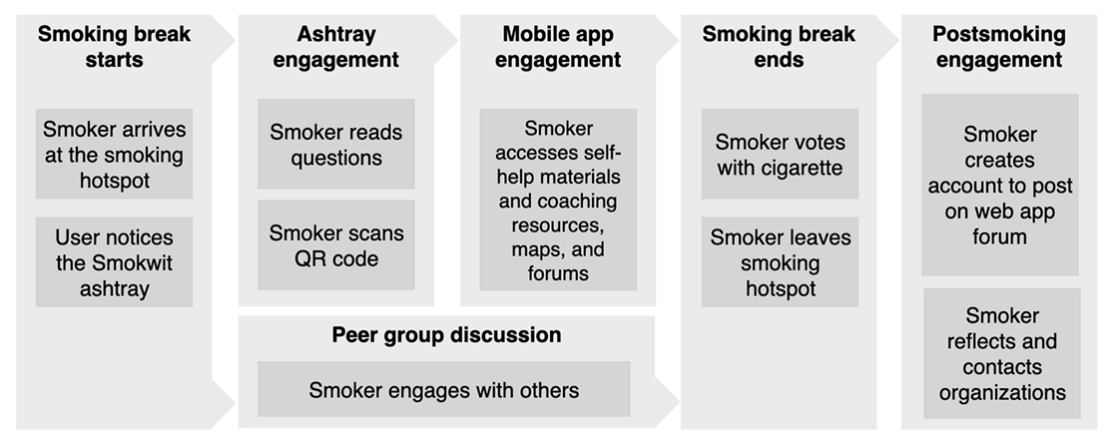
Smokwit smoker journey represented by 6 phases: smoking break starts, ashtray engagement, mobile app engagement, peer group discussion, smoking break ends, and postsmoking engagement.

At the center of the Smokwit system are connected voting ashtrays, which act as ambient transition devices, providing a link between the physical smoking hotspot and a mobile app, which provides digital support (Figure 2). The interaction with the Smokwit system can be described through 6 phases, representing a typical user flow (Figure 3). The journey begins when a smoker arrives at a hotspot and notices the connected ashtray during the “smoking break starts” phase. In the “connected ashtray engagement” phase, the smoker interacts with the ashtray by reading reflective questions and scanning a QR code to access additional digital resources, linking the physical and digital components of the system. The ashtray presents a question (Table 1) along with 2 answer options, each corresponding to a separate ashtray compartment. Smokers cast their vote by placing their cigarette butt in the slot, which reflects their answer. The questions were divided into 2 sets. The first set aimed to capture individual smokers’ perceptions, while the second set presented aggregated feedback from the broader smoking community on each campus. At the hotspot, smokers could also engage in peer discussions fostered by the ashtray’s presence, creating opportunities for shared reflection and mutual support. Scanning the QR code transitions the smoker to the “mobile app engagement” phase, where they can access self-help materials, coaching resources, maps, and forums. The journey may continue into the “postsmoking engagement” phase, where smokers further interact with the mobile app. They can create an account to participate in forums, analyze personal statistics, or contact support organizations.

**Table 1:** Poll questions displayed on Smokwit ashtrays and the number of cigarette butts collected for each response from 1 hotspot.

Poll question examples	Set	Option 1	Butts for option 1, n	Option 2	Butts for option 2, n
I smoked this cigarette to give myself a boost	1	Yes	51	No	82
I found this cigarette ...	1	Great	67	Not great	30
This cigarette will help me focus	1	I think so	32	No need!	22
On Campus 1, what % of cigarettes are smoked to get a boost?	2	<50%	20	≥50%	41
What % of Campus 1 smokers actually enjoy cigarettes?	2	<50%	5	≥50%	16
On Campus 1, what % of cigarettes are smoked to help focus?	2	<50%	10	≥50%	18

One of the main system design priorities for the user experience was lowering friction. We focused on the solution’s simplicity to ensure that anyone, regardless of prior experience or technical proficiency, can become a potential user. To engage users with self-help materials and group discussions, the system was designed following Zhang’s [25] 5 key principles for creating motivational affordances, which are design elements intended to promote specific user behaviors. These design principles (DPs) are (DP1) allowing autonomy and self-control, (DP2) supporting competence and achievement, (DP3) fostering social connections, (DP4) facilitating leadership and followership, and (DP5) triggering emotions. Moreover, the system design incorporates the Fogg Behavior Model [58], ensuring that all 3 essential elements as in motivation, ability, and behavior triggers are present to support behavior change in cigarette addiction effectively.

The Smokwit ashtray’s simple design eliminates the need for advanced abilities or complex instructions, ensuring that the system is intuitive and accessible to all users. Smokers can cast their “vote” by placing their cigarette butt in the compartment, which corresponds to their answer (DP1). This low physical effort supports the ability component [58] by minimizing barriers to engagement while simultaneously strengthening motivation by fostering a sense of autonomy and ease of use. Users are free to choose whether to participate and which ashtray they want to interact with. The system is designed to support competency and achievement (DP2) through a check-in mechanism where scanning a new ashtray’s QR code unlocks it in the mobile app and grants access to its statistics and forum. The app displays both discovered and locked ashtrays on a campus map and presents poll results, giving users a clear sense of progress. This feature is designed to motivate users to go to different places and engage in reflection both in person and within the app’s forum (DP3). This incorporates facilitator triggers, which reduce friction with spark triggers such as gamified rewards and visible progress to prompt participation in discussions [58]. When joining discussions, users create a username and can upload an avatar; however, their true identity is not required. Conversations are anchored in the ashtray’s shared question and poll results, offering opportunities for asynchronous peer reflection and social connections. The system also provides experience points (XPs) to users who participate in forum discussions to support the leadership or followership, social comparison, aspect (DP4). These points are displayed under the usernames in the forum, making active users more visible to others and potentially encouraging further engagement through subtle social referencing. This feature incorporates motivational elements such as social acceptance [58]. Finally, the system supports curiosity (DP5) with a bright yellow ashtray that draws attention, a rotating set of reflective questions on its display, and prompts to discover other ashtrays on campus. These features initiate a pleasure motivator, a spark trigger, which both arouse interest and motivate users to engage further [58].

#### Connected Ashtrays

**Figure 4:**
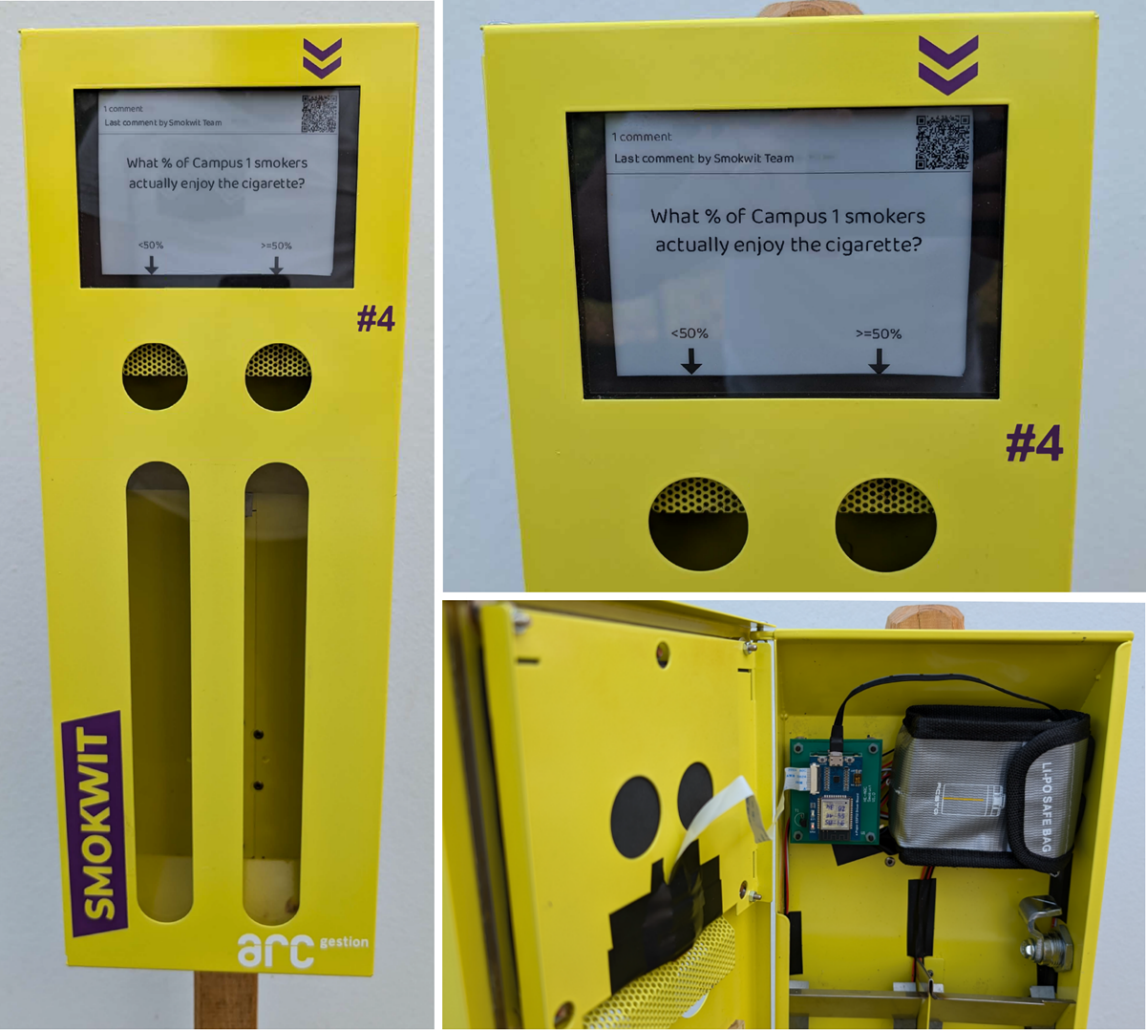
Smokwit connected ashtray at the smoking hotspot.

The Smokwit connected ashtrays (Figure 4) are based on modified commercial voting ashtrays. A voting ashtray consists of 2 compartments, each labeled with a distinct choice in the form of a printed question or poll. In the Smokwit version of the ashtrays, the paper has been replaced with a connected e-paper screen, and sensors were installed on the inside of the ashtray to allow the counting of cigarette butts in both ashtray compartments. An ESP32 microcontroller unit is used to pilot the e-paper screen, sensors, and Wi-Fi connectivity to (1) update questions remotely and (2) send counted votes to the mobile app. To make the ashtrays energy autonomous, they include a 10,000 mAh battery and a 6 W solar panel.

#### Mobile App

**Figure 5:**
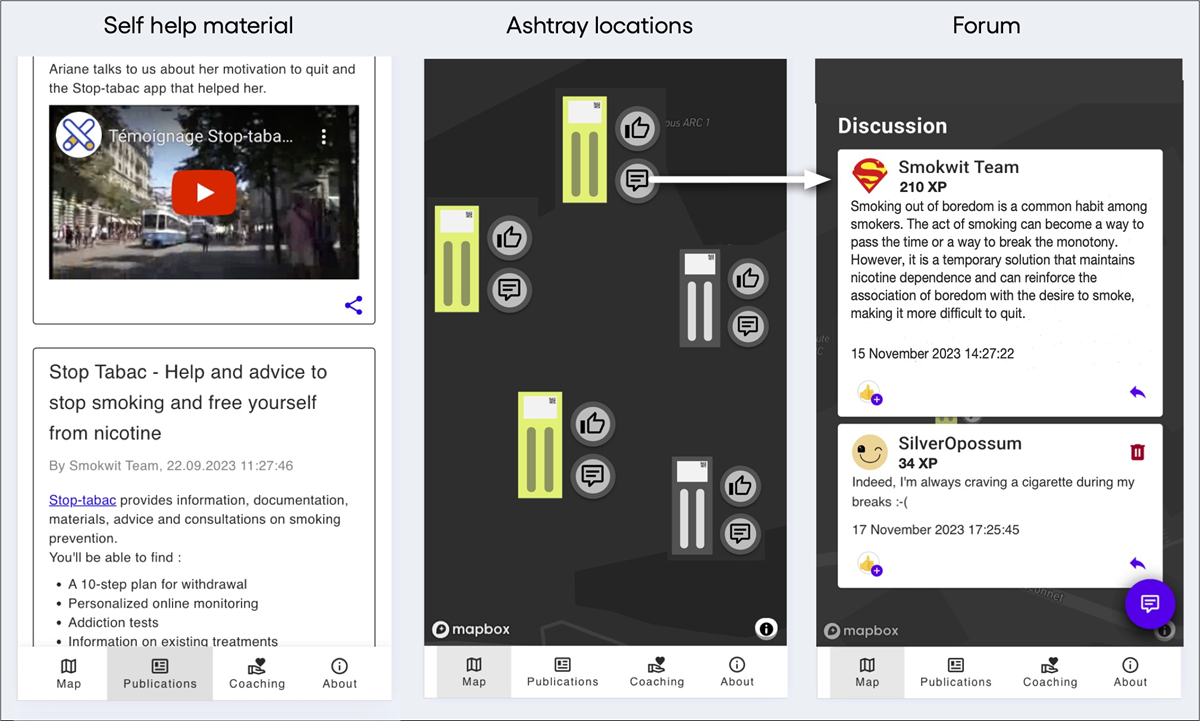
Smokwit mobile app with views of self-help material, a map showing connected ashtrays, and an example of the digital discussion associated with a poll question.

The Smokwit mobile app (Figure 5) was developed as a responsive web app. The mobile app provides self-help material with tobacco prevention content as well as a coaching section, where users can contact regional smoking cessation experts. The main screen of the mobile app is the campus 1 map, on which the Smokwit ashtrays have been geolocated. Each ashtray has a scannable QR code. By scanning the QR code, the user unlocks the ashtray on the mobile app and earns XPs. The physical ashtray can be used independently; however, the app enhances the experience by providing additional insights. When scanning an ashtray, it unlocks it on the map by becoming yellow. By unlocking the ashtray on the platform, the user directly arrives at the ashtray page and has access to the digital discussion forum associated with the ashtray’s current poll question. Indeed, users can access this information without registration. Open access promotes inclusivity and user engagement, making it easier for individuals to join in forum discussions and explore resources at their own pace. The user is invited to comment on the poll question by leaving a message or using emojis in the dedicated community section. Each action is rewarded with XPs. Posting and reacting to messages require an account, which can be created on the go by creating only a username and password. A username is automatically generated and suggested to the user to minimize friction and maintain anonymity. When the ashtray is unlocked, the user can also consult information regarding current and past polls as well as additional statistics such as the daily and hourly inflow of cigarette butts. There is an ashtray page for each Smokwit ashtray.

### Field Study

A field study was designed in the wild on 2 university campuses to assess the impact of the Smokwit intervention on enhancing smokers’ progress in their quitting process through their RTQ (hypothesis 1) by fostering self-reflection (hypothesis 2a) and peer support (hypothesis 2b).

#### Measures

To better understand the participants, in addition to age and sex, we also inquire about the age the smokers started, if they were smoking daily, and their number of previous attempts to stop smoking as a preliminary measure of their desire to stop [59,60].

To assess the user experience and understand self-reflection and peer support processes, we log activity on the mobile app as well as the number of votes from the ashtrays. Furthermore, to obtain more in-depth insights, we conducted semistructured interviews with smokers exposed to Smokwit and experts from smoking cessation organizations.

The selected metric to measure progress on the quitting smoking process is the RTQ ladder [59] (Table 2). This provides a graphic representation of the continuum of change, ranking smokers’ RTQ smoking from 1 to 10. Level 1 for smokers having no interest in quitting at all to level 10 for smokers who have become permanent nonsmokers and who have little concern that they will relapse. Levels 2 to 5 are precontemplators, according to the TTM. Level 6 is equivalent to the contemplation stage. Levels 7 and 8 indicate that the smoker is, respectively, in the preparation and action stages, whereas level 9 indicates maintenance.

**Table 2:** Readiness to quit ladder with stages of change, their level equivalents, and descriptions.

Stage of change	Level	Description
Termination	10	I have quit smoking, and I will never smoke again.
Maintenance	9	I have quit smoking, but I still worry about slipping back, so I need to keep working on living smoke-free.
Action	8	I still smoke, but I have begun to change, for example, cutting back the number of cigarettes I smoke. I am ready to set a quit date.
Preparation	7	I definitely plan to quit smoking in the next 30 days.
Contemplation	6	I definitely plan to quit smoking in the next 6 months.
Precontemplation	5	I often think about quitting smoking, but I have no plans to quit.
Precontemplation	4	I sometimes think about quitting smoking, but I have no plans to quit.
Precontemplation	3	I rarely think about quitting smoking, and I have no plans to quit.
Precontemplation	2	I never think about quitting smoking, and I have no plans to quit.
None	1	I enjoy smoking and have decided not to quit smoking for my lifetime. I have no interest in quitting.

#### Evaluation Setup

#### Overview

We conducted the field study on 2 university campuses following a quasi-experimental design with a treatment (campus 1) and a control (campus 2) condition. We performed the study in a campus setting because the student population would, by design, consist of young adults, who are our target demographic. Furthermore, in such contexts, students tend to smoke at the same time, meaning during lecture breaks, which is prone to group discussion. It is a quasi-experiment, as the participants were the natural members of each geographically distant campus of the same university and thus were not randomly assigned. Table 3 gives an overview of the field study.

**Table 3:** Experimental phases with study groups and participant amount.

	Phase 1 (June to September 2023): presurvey, n (%)	Phase 2 (September to December 2023): intervention	Phase 3 (January to March 2024): postsurvey, n (attrition %)	Phase 4 (June to November 2024): postinterviews, n (%)
Treatment	47 (65)	Prevention day + 4 Smokwit ashtrays	27 (43)	10 (21)
Control	25 (35)	Prevention day	19 (24)	—^a^
Experts	—	—	—	7 (—)
Total	72 (100)	—	46 (36)	10 (—)

^a^Not applicable.

#### Phase 1: Recruitment Pretest

Before the experiment began, we recruited participants by asking smokers in various smoking locations on the 2 campuses for their email addresses. A total of 119 participants registered for the study, and 72 fully completed the presurveys (47 for the treatment and 25 for the control conditions). Two researchers developed the survey and pretested it in collaboration with regional smoking cessation experts (Cipret Neuchâtel). It included 9 items on demographics, smoking habits, and the literature-validated RTQ scale, which participants completed in approximately 6.50 (SD 7.08) minutes.

#### Phase 2: Intervention

**Figure 6:**
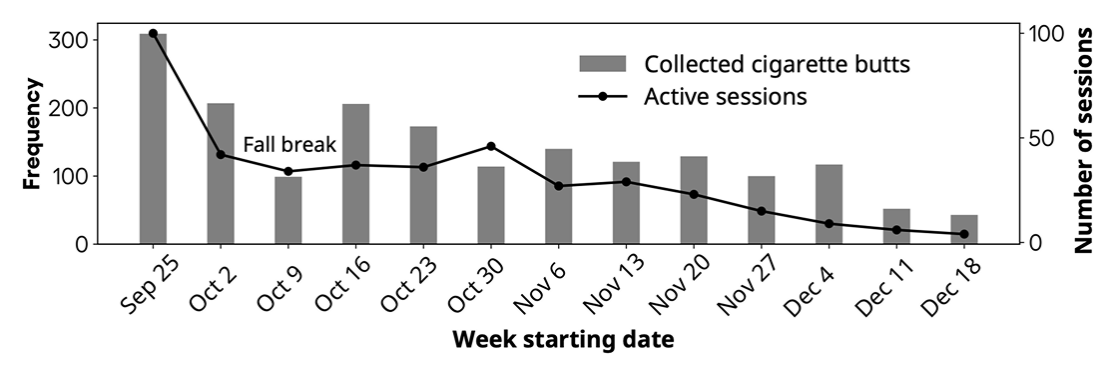
Number of cigarette butts collected by ashtrays and number of active sessions on the mobile app.

The intervention phase lasted 3 months. It began with a smoking prevention day on both campuses, led by 2 experts from a local smoking prevention organization (Cipret Neuchâtel). The event featured a free information stand aimed at encouraging participants in both conditions to consider quitting and providing contact information. This setup was designed to reduce the risk of social desirability bias by giving participants on both campuses a sense of being part of an experiment. During the entire intervention phase, 4 Smokwit ashtrays were deployed only on campus 1 (the treatment condition). Each Smokwit ashtray showed a different poll question, and the polls were updated every 3 days. In total, 70 poll items were created. The first set of 35 poll questions (ie, set 1 in Table 1) aimed to gather community data (eg, “I smoked this cigarette to give myself a boost,” yes or no). The second set of 35 poll questions (ie, set 2 in Table 1) aimed to raise awareness about consumption and encourage contemplation on quitting smoking (eg, “On Campus 1, what % of cigarettes are smoked to get a boost?,” <50% or ≥50%). Every butt tossed into a Smokwit ashtray was detected by sensors and considered for the poll question statistics. Figure 6 illustrates the number of cigarette butts tossed weekly alongside the number of sessions on the mobile app. These data indicate that smokers were more active in the early weeks of September. Reduced smoking activity happened when the weather gets colder, and this has been observed previously [61].

#### Phase 3: Postsurvey

The postsurvey included the same questions on smoking habits and RTQ as presurvey. A survey was sent to all participants after the experiment in mid-January 2024, with a follow-up email in late February. The postsurvey was fully completed by 46 valid participants who also completed the presurvey (27 in the treatment condition and 19 in the control condition). This took on average 5.30 (SD 2.06) minutes to complete.

#### Phase 4: Postinterviews

We conducted 2 rounds of follow-up interviews with participants to understand user experience and long-term impressions. The first round was conducted in June 2024 with 5 participants, and the second one in November 2024 with 5 additional participants. We invited participants who had completed both pre- and postsurvey and expressed interest in a follow-up interview. Through these interviews, we sought to understand motivations, barriers, and decision-making processes, providing deeper insight into their behavior and perceptions of the system. Furthermore, in November 2024, we interviewed 7 smoking cessation experts to gather their professional feedback.

## Results

### Overview

**Figure 7:**
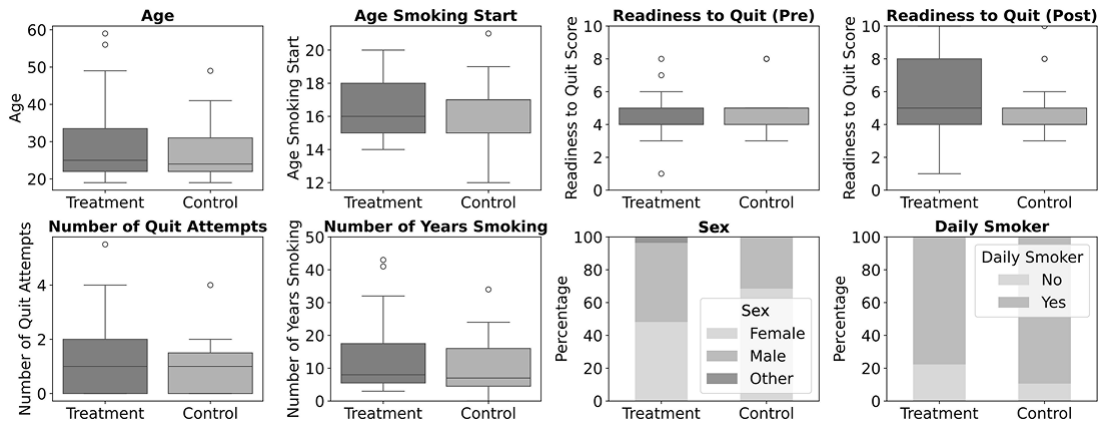
Presurvey results for demographics, smoking habits, and readiness to quit (treatment group: n=46 and control group: n=27).

Hereafter, we present the quantitative results of the quasi-experiment. We conducted all analyses using Python scripts, which are available alongside the datasets in Multimedia Appendix 1. The demographics and smoking habits are presented in Figure 7 and summarized in Tables 4 and 5. They show that the participants belong to the demographic that we planned to address, namely, mainly young adults in their 20s, who are in their precontemplation phase when it comes to stopping smoking.

**Table 4:** Summary statistics for treatment and control groups (n=46) collected in presurvey from participants who completed both pre- and poststudy surveys.

Variable	Treatment (n=27), n (%)	Control (n=19), n (%)
Female	13 (48)	13 (68)
Other	1 (4)	0 (0)
Daily smoker	21 (78)	17 (89)

**Table 5:** Descriptive statistics for age, age smoking start, number of quit attempts, readiness to quit (RTQ) in presurvey, and RTQ in postsurvey for treatment and control groups.

Variable	Treatment group			Control group		
	Mean (SD)	Range	25%, 50%, 75%	Mean (SD)	Range	25%, 50%, 75%
Age (years)	30.00 (11.73)	19-59	22, 25, 33	27.37 (8.72)	19-49	22, 24, 31
Age smoking start (years)	16.59 (1.74)	14-20	15, 16, 18	16.42 (2.06)	12-21	15, 17, 17
Number of quit attempts	1.52 (1.47)	0-5.5	0, 1, 2	0.89 (1.10)	0-4	0, 1, 1.5
RTQ (pre)	4.37 (1.36)	1-8	4, 4, 5	4.95 (1.51)	3-8	4, 5, 5
RTQ (post)	5.67 (2.62)	1-10	4, 5, 8	5.00 (1.83)	3-10	4, 4, 5

In terms of interaction with the Smokwit system, the QR codes on the ashtrays were scanned 245 times. Based on session IDs, we could account for 74 people with an average interaction duration of 2 minutes and 11 seconds (SD 70 seconds). The ashtrays’ webpages were viewed 2409 times, with a mean of 50.9 (SD 35.0) views per user and an average length of engagement of 59 (SD 36.1) seconds. The page containing self-help material was viewed 44 times, with a mean of 3.67 (SD 1.57) views per user and an average length of engagement of 20 (SD 7.54) seconds. This use is in line with what could be expected with the number of participants in the treatment group (77 people in the beginning and 46 at the end).

### Attrition Rate

The overall attrition rate of the study was 36% (n=46), with 24% (n=19) in the control group and 43% (n=27) in the treatment group. This attrition rate is rather high and common in smoking cessation studies over several months, where attrition rates have been found to be as high as 77% [62] and even 90% [37]. Nevertheless, it is important to understand that this attrition might have created an imbalance in the sample and potentially introduced bias. We assessed if there was a statistical difference between participants who provided full answers to both surveys (n=46) and those who only answered the presurvey (n=72) across all measures taken in the presurvey (sex, age, age smoking start, group, number of quit attempts, and RTQ). The sample was balanced, although 2 motivation-related variables showed marginally significant outcomes. Smokers with more quitting attempts (t_25_=1.81; *P*=.08) and higher RTQ scores (t_25_=1.79; *P=*.08) may have left the study more often than those with lower RTQ scores and fewer attempts to quit. The results suggest that, even if such imbalances exist, the primary target population of this study, namely, individuals in the early stages of their smoking cessation process, has been retained in the sample. Nevertheless, future studies with larger samples should replicate our findings to ensure that attrition bias did not modify the results.

### Ambient Gamification and RTQ (Hypothesis 1)

To test hypothesis 1, we analyzed whether the Smokwit intervention increased participants’ RTQ compared to a control group. A linear mixed effects model was used with restricted maximum likelihood estimation [63]. The model included time (pre or post), treatment (Smokwit vs control), and their interaction, along with relevant covariates: sex, age, number of quit attempts, age smoking start, and smokes daily. A random intercept was included for each participant to account for individual differences in baseline RTQ.

**Figure 8:**
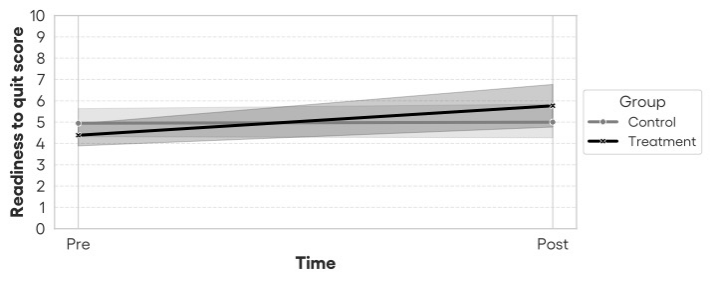
Visual representation of the interaction effect between time and group on readiness to quit scores with 95% CIs.

The results (Table 6) show a possible interaction effect (Figure 8) between time and treatment: when controlling for all other variables, participants in the treatment group showed a potential greater increase in RTQ scores compared to the control group after the 3-month experiment (b*=*1.33; *z*=1.91; *P*=.06). Even though the result is close to statistical significance, it does not reach it. This finding hints at possible support for hypothesis 1. The effect size for this interaction, measured using Cohen *f*^2^, was 0.04, representing a small effect size according to conventional benchmarks [64]. While modest, such effects are typical and meaningful in behavioral science and digital health research [65]. To evaluate model fit, we computed the marginal and conditional *R*^2^ values. The marginal *R*^2^ (variance explained by fixed effects) was 0.27, and the conditional *R*^2^ (fixed + random effects) was 0.38, indicating that both predictors and individual variability contributed meaningfully to the variance in RTQ. The results indicate no significant association between RTQ and sex or the age of smoking start.

Additionally, the results show that the number of past quit attempts is positively associated with RTQ (b=0.60; *z*=3.81; *P*<.001), and daily smokers reported lower RTQ scores than nondaily smokers (b=–1.07; *z*=–1.99; *P*=.047), meaning that the higher the number of previous quit attempts, the higher the smoker’s RTQ, and the more a smoker smokes, the lower their RTQ. Furthermore, the results also show a negative marginally significant trend (b=–0.04; *z*=–1.83; *P*=.07), indicating that younger smokers may have a higher RTQ than the older ones.

Note that the results also indicated a potential imbalance in the baseline sample when controlling for the other variables, because the main effect of treatment was significant (b=–1.09; *z*=–1.97; *P*=.049) when pooling pre- and postresults together. This means that the treatment group may have had a lower baseline RTQ than the control group. Figure 8 visualizes these results.

To further investigate the difference between pre- and postlevels of RTQ in both conditions, we conducted a complementary paired sample for each group. In the treatment group, RTQ scores increased significantly from pre- to postintervention (pre: mean 4.37, SD 1.36; post: mean 5.67, SD 2.62; t_26_=2.72; *P*=.01), with a medium effect size (Cohen *d*=0.52). Conversely, the control group showed no significant change (pre: mean 4.95, SD 1.51; post: mean 5.00, SD 1.83; t_18_=0.11; *P*=.91; *d*=0.03).

A post hoc power analysis based on the observed effect size (*d*=0.52) and sample size (n=27) indicated a power of 0.74 to detect the change in RTQ, which is close to the conventional threshold of 0.80 [64]. This suggests that while the study may be slightly underpowered, the observed effect is meaningful and warrants further investigation with larger samples.

Taken together, these results cautiously support hypothesis 1: ambient gamification through the Smokwit device may help increase RTQ among young adults; however, future studies with larger samples are needed to confirm the robustness of this effect.

**Table 6:** Results of the mixed linear model regression on readiness to quit scores.

Effect	Coefficient b (SE)	*z* score	*P* value	95% CI
Intercept	7.69 (2.03)	3.78	<.001	3.71 to 11.66
Time (post)	0.05 (0.53)	0.10	.92	–0.99 to 1.09
Treatment	–1.09 (0.55)	–1.97	.049	–2.16 to –0.01
Treatment over time (interaction)	1.33 (0.70)	1.91	.06	–0.03 to 2.70
Number of quit attempts	0.60 (0.16)	3.81	<.001	0.29 to 0.91
Age of smoking start	–0.09 (0.11)	–0.81	.42	–0.30 to 0.13
Smokes daily	–1.07 (0.54)	–1.99	.047	–2.12 to –0.02
Sex (male)	0.68 (0.42)	1.63	.10	–0.14 to 1.49
Age	–0.04 (0.02)	–1.83	.07	–0.08 to 0.00
Group variance	0.45 (0.34)	—^a^	—	—

^a^Not applicable.

### Qualitative Analysis (Hypothesis 2a and Hypothesis 2b)

#### Overview

To gain additional insights into the user experience with Smokwit, we conducted interviews with 10 participants and 7 smoking cessation experts. We conducted semistructured interviews with 10 participants (Participant 1-Participant 10) from the treatment group between 6 and 9 months after the end of the study. These interviews focused on four key themes: (1) interactions with the ashtrays, (2) self-reflection, (3) peer support, and (4) user feedback. The discussions explored participants’ memories of the study, including their interactions with the ashtrays, the system, and other smokers, as well as their smoking habits and suggestions for improving the intervention. Additionally, we presented our project at the Swiss Association for Tobacco Control (AT Switzerland) Conference 2024, where we collected feedback from 7 smoking cessation experts (Expert 1-Expert 7) on our system.

We followed a qualitative data analysis via a general inductive approach [66]. Interviews were audio-recorded, transcribed uniformly, and reviewed multiple times to identify recurring patterns and derive significant themes.

#### Ashtrays Interactions

The interviews revealed that participants found the Smokwit ashtrays’ questions engaging, particularly at the beginning of the intervention. Participant 1 remarked, “I liked these questions, not all of them, but often, they were funny. They made me laugh,” highlighting the positive reception to the reflective prompts. Participant 3 also described the questions as highly engaging and innovative, noting how they encouraged smokers to reflect on their habit. Similarly, Participant 4 appreciated the weekly changing questions, which sparked curiosity and led to meaningful reflections on smoking habits. The gamified and humorous design of the questions was central to sustaining participants’ interest and stimulating self-reflection. Participant 5 highlighted the ashtrays’ visibility, noting their “super visible yellow” color, which made them accessible and noticeable on campus. Participant 9 and Participant 8 also remarked on the ashtrays’ appealing design, describing it as “hard to miss” and “innovative and engaging, especially during the initial weeks of use.” The ashtrays managed to attract attention beyond smokers, as Participant 3 observed, “We were all going to see the question, even non-smokers were going to see.” However, this broad appeal diminished over time as the novelty wore off, as Participant 3 noted, “At first, it was attractive, but afterward, I think they didn’t change the questions every day.” Practical challenges, such as crowding during peak hours, also reduced engagement, as Participant 10 explained, “The voting process sometimes felt crowded, which could have discouraged participation.” Experts empathized with the nonintrusive nature of the ashtrays, noting their passive presence in smokers’ environments. Expert 2 remarked, “I like the passive side of the object. It is present in the smoker environment, but it is not intrusive,” which allowed participants to engage without feeling pressured. Expert 1 highlighted the ashtrays’ potential for workplace deployment, suggesting that companies could use them to gather data and justify targeted interventions. Expert 6 further noted, “Smoking prevention centers would be able to use these ashtrays as an extra tool prior to traditional in-person meetings and hold discussions on various subjects depending on the information gathered.” Expert 7 recommended testing the ashtrays in public spaces such as bus stops to evaluate their effectiveness in diverse contexts, “It could be interesting to test it on bus stops also.” These suggestions highlight the flexibility of intervention design for alternative environments. Despite challenges such as diminished novelty and logistical issues, the intervention successfully sparked curiosity and reflection among participants. The ashtrays’ design and passive approach made them a versatile tool, with potential apps extending beyond individual behavior changes to organizational and community contexts.

#### Self-Reflection

The interviews revealed that the Smokwit system fostered self-reflection, disrupting habitual smoking behavior. The questions posed by the ashtrays prompted smokers to think more critically about their habits. As Participant 1 remarked, “it (ashtray) makes you think, yes, think about what you’re doing,” while Participant 2 emphasized, “It makes smoking more conscious.” These prompts heightened awareness and occasionally sparked discussions about the broader implications of smoking, as observed by Participant 10. Participant 4 highlighted the intervention’s dual impact, stating, “It’s true that these are not necessarily reflections that we usually ask ourselves.” By encouraging such reflections, the ashtray helped participants reconsider their behaviors and the broader implications of their smoking habits. Participant 3 acknowledged, “It (ashtray) told me even more that I should quit smoking,” suggesting that while the ashtrays did not directly reduce smoking, they reinforced the motivation to stop. This indicates that the intervention planted seeds of change, providing a foundation for future behavior modification. The gamified aspect of the ashtrays, such as combining playful and thought-provoking questions, further stimulated self-reflection. As Participant 10 remarked, “I liked the fact that it was a bit playful. And then, it made us think, so they were surely good questions.” However, Participant 6 noted that without frequent updates, the questions became less relevant over time. Participant 2, who initially felt uncomfortable with the ashtrays’ focus on her smoking habit, engaged indirectly through discussions with colleagues who had interacted directly with the system. Even by indirect engagement, her smoking became more conscious, demonstrating the ashtrays’ broader impact on mindfulness. Although participants reported limited immediate changes in their smoking behavior, the intervention reinforced awareness and the desire to quit. Experts emphasized the ashtrays’ ability to disrupt smoking rituals, prompting deeper reflection. Expert 3 explained, “Smoking is seen by smokers as a ritual. Intervening during the ritual is absolutely fantastic and has, in my opinion, great potential. It also breaks the routine.” External stressors, such as life challenges and academic pressure, influenced participants’ engagement with reflective prompts. Participant 3 mentioned going through a particularly difficult period, which may have impacted both smoking habits and engagement with the ashtrays: “We’ve always got excuses, so you had put them (ashtrays) in July, you had put them in whenever. It would all be the same. I had a lot of problems at that time, it would not change anything,” highlighting the impact of personal difficulties on the intervention’s effectiveness. Participant 7 similarly emphasized, “Quitting is harder than reflecting, it’s the execution that counts.” Additionally, Participant 5 pointed out that academic stress often led students who did not normally smoke to resume during high-pressure periods, such as examinations. These insights suggest that while immediate behavior change was limited, the intervention laid the foundation for future efforts by increasing awareness and prompting critical self-reflection. By encouraging smokers to think more critically about their habits, even amid external stressors, the Smokwit system shows promise as a long-term catalyst for change. These findings provide some qualified support for hypothesis 2a.

#### Colocated Peer Support on Smoking Hotspots

Our results show that the Smokwit system fostered peer support and facilitated meaningful discussions among smokers. The intervention encouraged conversations about smoking cessation, helping smokers build a support network. Participant 1 shared, “We talked a little about our experiences. Of our attempts to quit,” illustrating how the ashtrays encouraged smokers to share their personal experiences and reflections. The gamified elements of the ashtrays, such as the changing questions and the visible, playful design, served as conversation starters, breaking down barriers and making it easier for smokers to engage with one another. According to Participant 10, the ashtrays “sparked meaningful debates among colleagues about smoking,” while Participant 9 noted, “The ashtrays encouraged casual debates among smokers, sometimes involving non-smokers too.” These interactions fostered a sense of community, as observed by Participant 4: “And then the interactions we can have with the smokers around.” Experts emphasized the strength of leveraging smoker communities for behavior change. Expert 3 explained, “A smoker community is felt by the smokers as an entity. Acting in the community itself is a real strength of the project. Smokers in the community will talk with each other and are influenced by each other.” Expert 6 suggested that the ashtrays could be deployed in workplace settings to facilitate discussions before formal interventions, stating, “Smoking prevention center could use these ashtrays to promote dialogue around smoking behaviors and cessation efforts prior to in-person meetings.” Expert 7 proposed that using ashtrays over an extended period before interventions would help target those most willing to engage in cessation programs. The ashtrays promoted social connections and collective reflection, creating a supportive environment, which encouraged participants to reconsider their smoking habits. Participant 4 mentioned that the ashtrays often led to small debates, reinforcing the collective effort to rethink smoking behaviors. Similarly, Participant 3 observed that both smokers and nonsmokers were drawn to the ashtrays, leading to some level of interaction and conversation, although this later declined as the novelty wore off.

By facilitating conversations and fostering social connections, the Smokwit system showed potential in influencing attitudes concerning smoking within the campus community. These findings suggest that the intervention not only supported individuals but also laid the groundwork for a more collaborative approach to addressing smoking habits, providing some qualified support for hypothesis 2b.

#### User Feedback

Participants offered valuable suggestions for improving the Smokwit intervention. Participant 1 and Participant 10 proposed extending the playful voting system using cigarette butts, which Participant 10 described as “a simple yet engaging mechanism.” Participant 9 and Participant 5 proposed expanding the forum functionalities by creating a dedicated space for smokers to log their reflections digitally, emphasizing that “a platform for shared experiences could boost long-term involvement.” Participant 9 suggested enhancing engagement by offering rewards following interactions with the ashtray, such as displaying live statistics not only within the mobile app but also directly on the ashtray screen. To keep the content relevant, Participant 3 highlighted the importance of updating questions more frequently and incorporating humor. Technological challenges with the app were sometimes a concern. Participant 6 and Participant 1 experienced difficulties scanning QR codes due to outdated smartphones, while Participant 7 and Participant 8 preferred direct interactions with the ashtray over using a QR code. Participant 7 noted, “Taking out the phone and scanning the QR code, even if it’s only two minutes, can be a hindrance,” and Participant 8 added, “Interacting directly with the physical object would be more immediate and engaging.” Experts recommended tailoring ashtray questions to different stages of behavioral change and integrating self-management tools. Expert 1 remarked, “It could be a good idea to have a similar kind of artifact for sugar and alcohol but also other forms of tobacco/nicotine consumption.” Expert 5 highlighted the value of deploying ashtrays in diverse settings, such as universities, to compare results across demographic groups, noting, “I would like to have this ashtray at the university where I work to compare the results between different types of cultural backgrounds.” Both participants and experts offered constructive feedback for improvement, offering a clear path for refining the Smokwit intervention. By addressing these technological opportunities and incorporating feedback, future iterations of the Smokwit intervention can become more engaging and impactful.

## Discussion

### Principal Findings

The results provide tentative support for the fact that including a gamified ambient object—a connected voting ashtray—at a smoking hotspot visited by young adults can complement existing evidence-based interventions and lead to increased RTQ among this demographic. Our findings align with previous research, which shows that simple interventions can potentially yield positive results [6,34]. Our controlled quasi-experiment results indicate a potential tendency for participants in the treatment condition to progress from the precontemplation to the contemplation stage, which could potentially lead to an increase in RTQ compared to the control condition (hypothesis 1). This tentative result may represent an early indication of the intervention’s potential to motivate change in young smokers, though its significance needs investigation in larger samples. While previous research has shown promise for in-person interventions at the smoking hotspots to leverage the act of smoking as an opportune moment to incentivize change [45], our research extends these findings by showing that ambient objects could be used as transitional devices. Although this study extended over 3 months and included follow-up interviews at 6 and 9 months, future research should aim to replicate and extend these findings in studies with longer durations and larger sample sizes. This would help clarify the role of novelty effects, the influence of community dynamics, and participants’ ongoing progress in the smoking cessation journey. Building on our work, future studies could also use path analysis to test the underlying conceptual model and examine both direct and indirect effects of the intervention components.

### Characteristics of the Target Population

The Smokwit intervention focused on young adults, aged 18 to 35 years, an important and understudied demographic. Indeed, smoking initiation often occurs before 21 years [17], and young adults tend to remain in the precontemplation stage longer due to lower perceived risks [45]. By embedding gamified ashtrays in habitual smoking environments, the intervention facilitated reflection and dialogue without requiring formal participation. The gamified intervention was tailored to young adults with gamification and fun poll questions, which were appreciated by participants. Although the study targeted young adults, participants older than 35 years of age were not excluded, reflecting the diversity of real-world smoking environments.

In our qualitative analysis, stress emerged as a key influence on smoking behaviors, with participants citing academic pressure and personal challenges as triggers. This is aligned with previous findings, which list smoking urges and stress as smoking triggers [67,68]. Participants often indicated that life challenges, such as academic or personal stress, played a role in their smoking patterns and their engagement with the intervention. Future work could aim to better understand the role of stress in the outcome of such interventions and how they could be designed to better cater to this characteristic.

### Ambient Interaction

Interaction analyses and interviews shed light on the mechanisms, which led to this outcome. First, the connected ashtrays, with their thought-provoking questions and gamified features, led participants to engage with the digital material by scanning the QR codes. The qualitative data suggest that the engagement with the ambient object played a critical role in raising awareness about smoking behaviors and encouraging self-reflection (hypothesis 2a). This aligns with previous research in other contexts, which showed the use of an ambient object to transition from physical to digital environments [24]. This also aligns with literature suggesting that behavior change apps should be designed to give users maximum control over their level of engagement and disengagement [69]. In this study, the Smokwit ashtray acted as a passive entry point into the digital intervention. Participants did not need to sign up or commit to a program to begin reflecting on their behavior. Instead, smokers encountered provocative, low-effort prompts in a familiar space, which encouraged casual interaction. This dynamic encouraged ongoing self-assessment, even among those not actively seeking to quit, and appeared to contribute to increased RTQ over time. This approach differs from typical behavior change apps, which rely on continuous digital engagement [70]. In Smokwit, a minimal physical interaction, voting with a cigarette butt, initiated a reflective process and offered a seamless path into digital support. Future research could investigate how to deepen these interactions directly at the smoking hotspot. For instance, integrating chatbots into the ashtray’s interface could increase playfulness and interaction while reducing friction to access self-help material [59]. Moreover, they could contribute to increasing emotional attachment to the system and potentially motivation to change behavior [71]. One participant mentioned that even though she felt the intervention was positive in the end, it could come across as moralizing. This could be problematic, as it might create reactance and backfire [72]. Future work should clarify how widespread this feeling is and how design strategies can mitigate unintended negative effects.

### Sense of Community

Our findings align with previous literature, supporting that engagement in digital smoking cessation communities is positively associated with the process of change [8]. While face-to-face peer groups have long been recognized as effective for fostering behavior change [32], this study extends that understanding by demonstrating the unique value of spontaneously formed, physically colocated peer groups. These groups emerge naturally at smoking hotspots, therefore eliminating the need for individuals to actively register or join formal programs. By delivering the intervention directly to smokers in their habitual environments, this approach reduces many of the typical barriers associated with participating in structured peer-support programs and leverages a gamified, spontaneous design to encourage engagement. Smokers may find it easier to make changes in their own lives if they are encouraged to share their experiences in digital communities dedicated to quitting smoking [8], but also in real-life, directly at the smoking hotspot. The ashtrays not only facilitated individual self-reflection but also promoted social interactions among smokers (hypothesis 2b). The gamified questions functioned as conversation starters, enabling smokers to discuss their experiences and challenges in real time, directly at the smoking hotspot. This sense of community fostered through the intervention may have reinforced participants’ RTQ by providing social support, an essential component in the process of behavior change [6]. Initiatives such as Weight Watchers have shown that “the magic of the meeting” has long been seen as an effective means of enhancing behavior [32]. Future work should explore how these spontaneous peer groups could be sustained over longer periods and investigate the mechanisms underlying their formation and effectiveness. Additionally, future research could examine the interplay between the various components of this intervention, including the app, forum, information, and ambient devices, as well as the influence of the implementation context. The context in which the intervention is deployed may mediate the sense of community it fosters, which is critical for its success [73].

### Implications for Practice

Smokwit facilitated discussions among campus smokers about the intervention and, to some extent, quitting smoking. The physical-digital integration, combining ashtrays at smoking hotspots with a mobile app, engages young smokers in both physical and digital environments. This approach increases accessibility and encourages spontaneous peer support by enabling interactions at familiar locations while providing digital resources and community forums without time and space limits. Moreover, it also improved the RTQ.

The findings from this study, informed by participants’ experiences and smoking cessation experts’ feedback, suggest several practical strategies for designing and implementing smoking cessation interventions. First, embedding interventions in habitual environments, such as smoking hotspots, fosters spontaneous peer groups. This approach encourages immediate social support and organic discussions about smoking behaviors. Second, tailoring intervention content to align with different stages of behavioral change enhances relevance and effectiveness, particularly when paired with gamified elements, which sustain engagement. To address varying user preferences, digital tools such as web platforms for sharing reflections can complement physical interventions and encourage long-term involvement. Experts highlighted the ashtrays’ passive and accessible design, which reduces barriers to participation and makes them suitable for diverse settings, including workplaces, bus stops, and public spaces. The ashtrays’ ability to collect data while engaging participants underscores their potential for broader applications in other contexts. These insights emphasize the importance of integrating interventions into users’ daily routines and combining physical and digital elements to maximize accessibility and impact. Future research could further explore how such an intervention could leverage context particularities to increase engagement. For instance, research could investigate if social mechanisms such as competition between groups or reciprocity could be used to foster higher peer support or a sense of community around a digital intervention. Such refinements could improve RTQ and deepen the intervention’s long-term impact.

### Limitations

This research is not without limitations. First, as with other experiments performed in the wild over several months, sample decline and potential differences between conditions (campus 1 and campus 2) might be beyond the researchers’ control. To address these sample differences, we controlled demographic characteristics such as age and sex in the analysis. However, other factors may still have influenced the outcome. Second, the intervention included several aspects of gamification, such as initial prompts (the poll questions), a treasure hunt feature (finding other ashtrays), and status (the XPs), making it challenging to isolate the effects of individual components. Third, while the ashtrays initially attracted significant attention, engagement declined over time. The reason for this cannot be clearly identified because it could be due to the waning of the novelty effect or lower smoking activity during cooler months to name 2 potential reasons. Future studies could aim to better understand optimal timing and durations for such interventions. Finally, external factors such as personal challenges and academic stress further moderate the intervention’s effectiveness on smoking behavior. The postintervention interviews demonstrated that the Smokwit ashtrays positively affect smokers’ awareness and social interactions regarding smoking. Although the immediate impact on smoking behavior was limited, the intervention raised consciousness concerning smoking habits and sparked discussions about quitting, providing a foundation for future enhancements, which could further amplify the intervention’s effectiveness and reach. It is important to note that the higher education context of the study may limit generalizability, because participants were more likely to adopt technology than the general population. Finally, the RTQ scale relied on self-reported data, which may be subject to social desirability bias. Additionally, the postsurvey timing at the beginning of the year, from January 17 to March 5, 2024, coincided with the “New Year’s Resolution” effect, which may have inflated RTQ scores. While these overestimations likely affected both conditions similarly, it cannot be excluded that one condition was more susceptible to these biases than the other.

### Conclusions

In this study, we investigated how a gamified ambient intervention called Smokwit deployed directly at a smoking hotspot could help smokers progress at the early stage of their smoking cessation process. This is a crucial public health issue, as this demographic represents the largest tobacco consumer base. Our approach was designed to complement existing evidence-based interventions and act as an entry point toward self-help material and peer support. We designed gamified voting ashtrays, which would engage smokers at the smoking hotspot and lead them to discuss with each other and access digital material, leading them to increase their RTQ. The qualitative results of our field experiment showed that the intervention did indeed foster digital interaction and discussions among peers. Furthermore, our quantitative analysis provided preliminary evidence that smokers in the intervention condition may have experienced a greater increase in RTQ, more than those in the control group, although the statistical strength of this observation remains limited. Overall, these findings are encouraging and indicate that well-designed digital interventions can potentially act as important complements to existing interventions, driving significant behavior change and promoting healthier lifestyles within the campus community. Despite encouraging trends, the study’s limited power necessitates cautious interpretation of the results. These preliminary findings should be confirmed in future trials with larger and more balanced samples.

## Appendix

Multimedia Appendix 1 Dataset and analysis scripts (Jupyter Notebook) for transparency and reproducibility.

## Abbreviations

DP: design principle
RTQ: readiness to quit
TTM: transtheoretical model
XP: experience point
