# Supplementary figures and images for: Gamified Physical-Digital Smoking Cessation Intervention for Young Adults: Mixed Methods Development and Usability Study
Source: JMIR Hum Factors. 2025 Sep 19;12:e72749. doi: 10.2196/72749 (PMC12495370; doi:10.2196/72749)

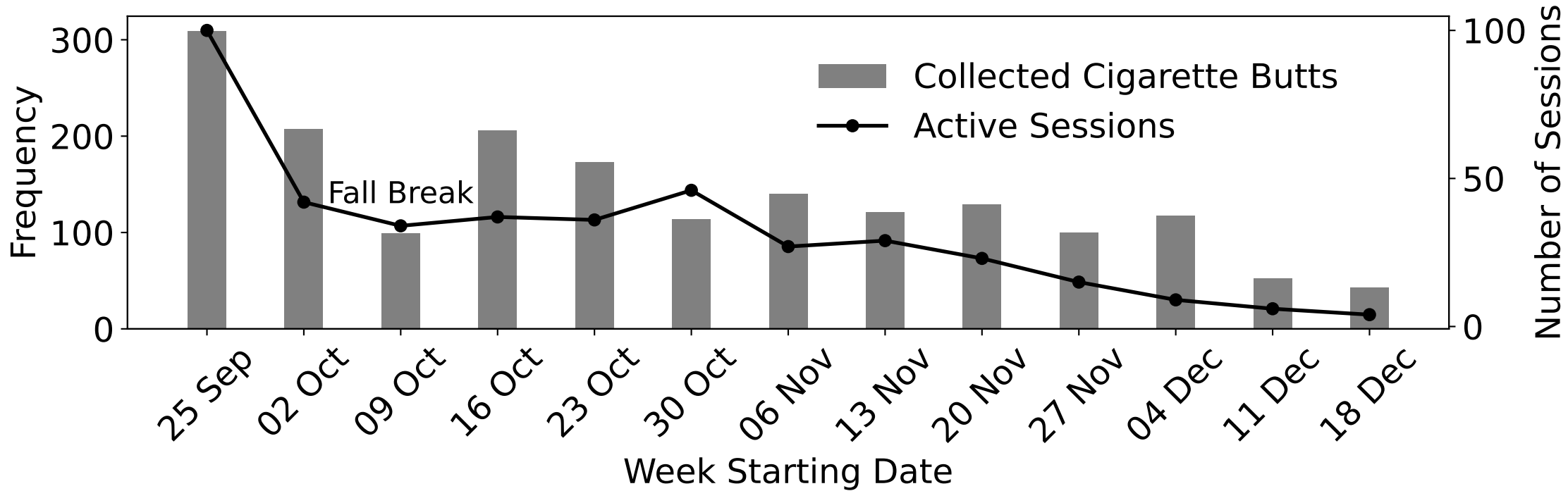

Supplement: Multimedia Appendix 1 [file humanfactors_v12i1e72749_app1.zip › supplementary material/activity.pdf]

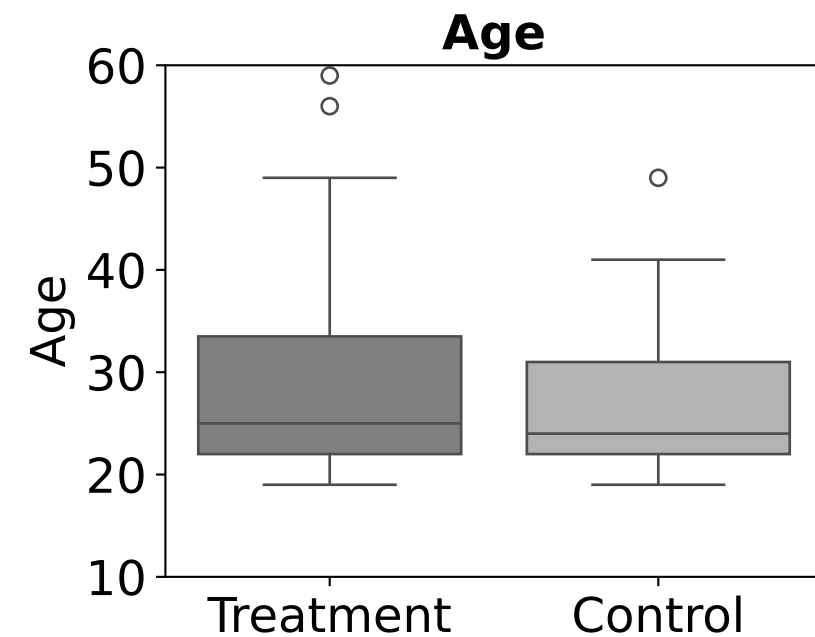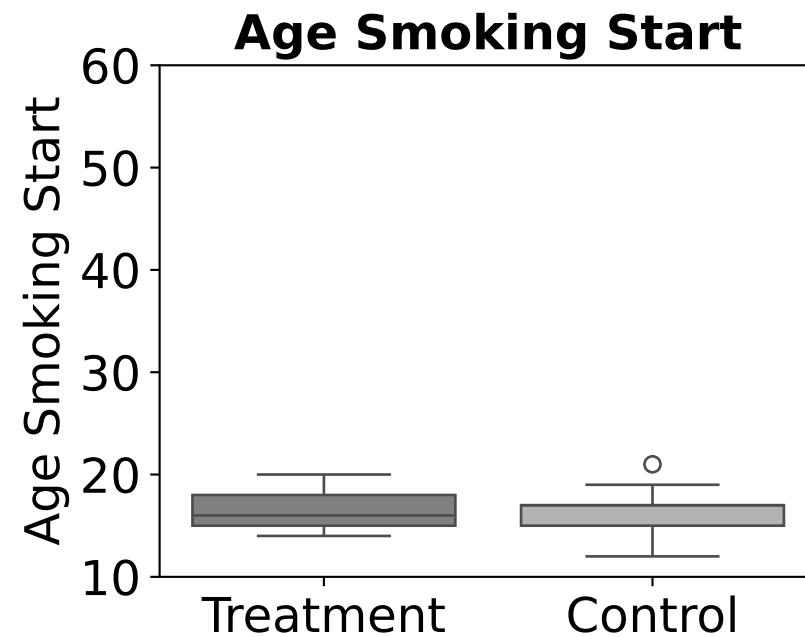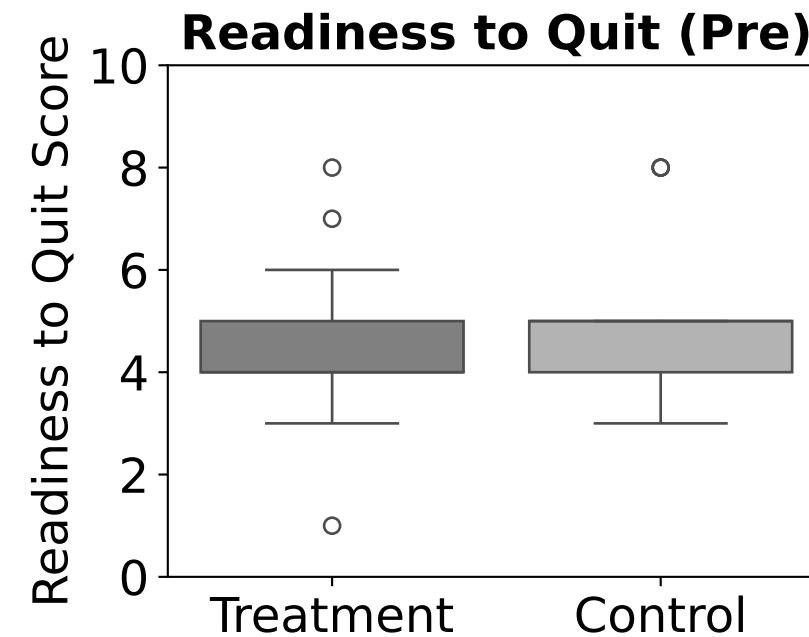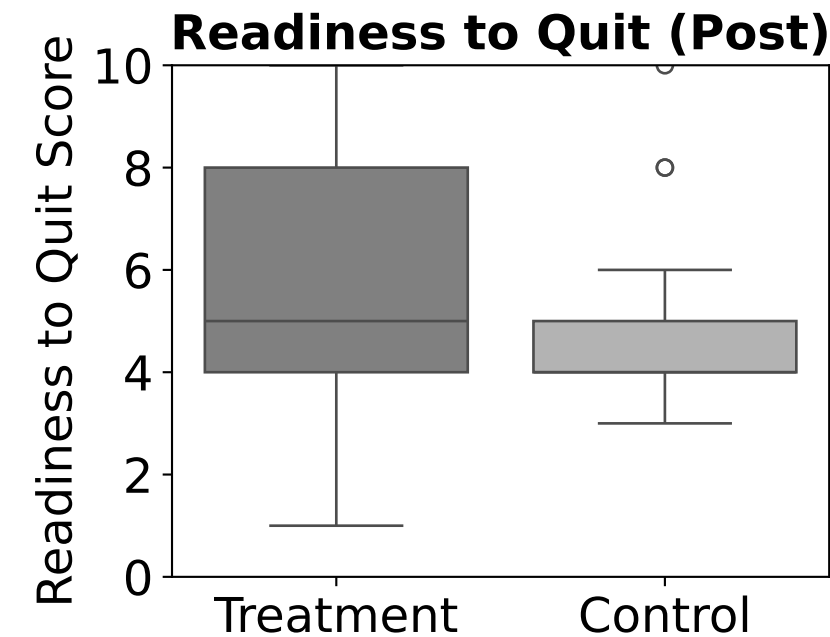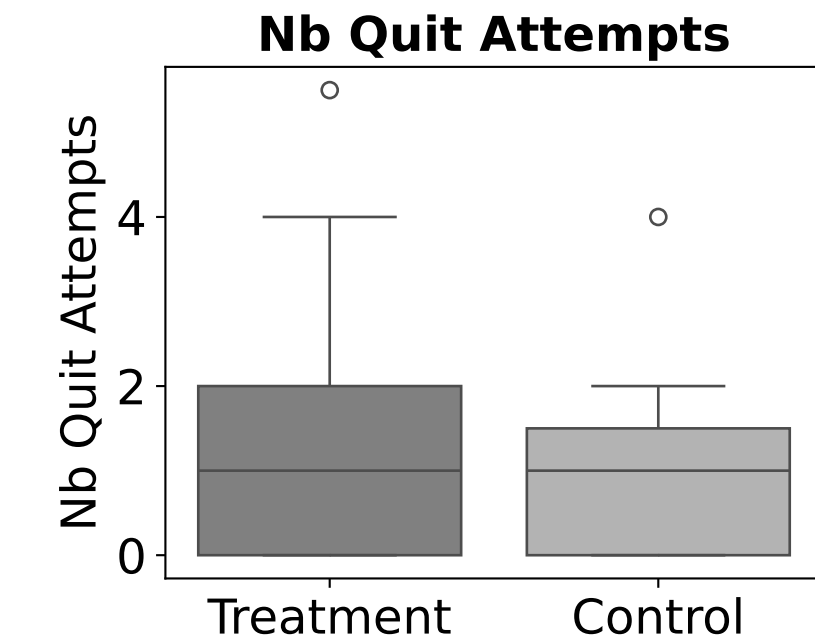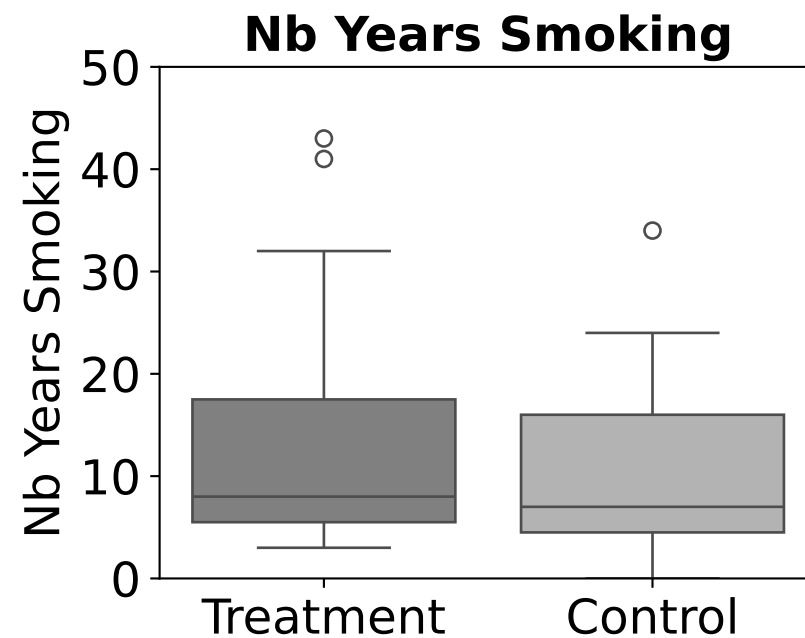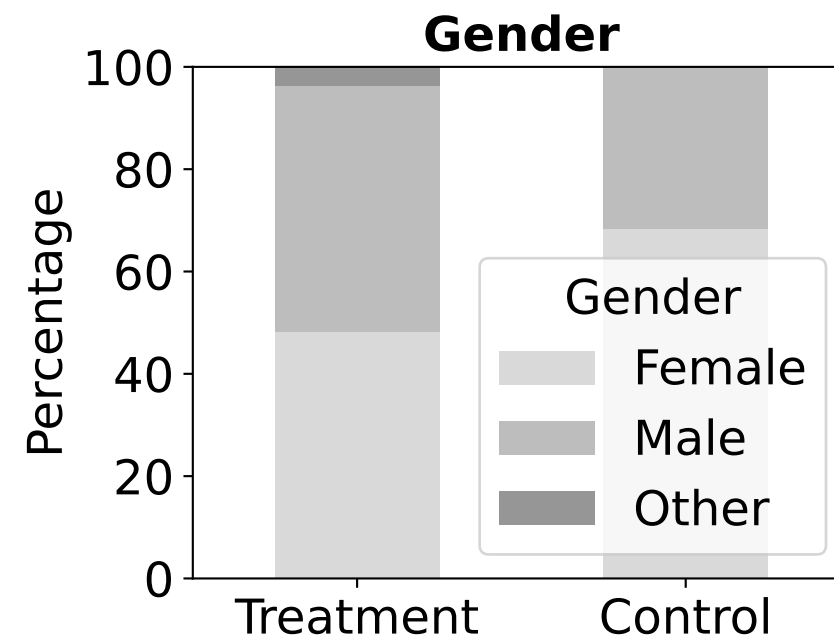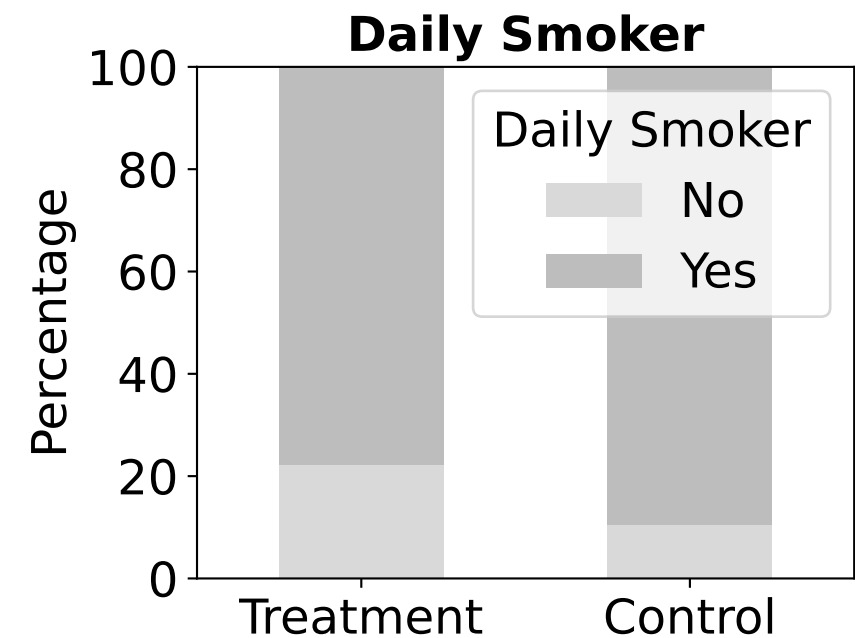

Supplement: Multimedia Appendix 1 [file humanfactors_v12i1e72749_app1.zip › supplementary material/combined_plots.pdf]
